# Experiences of criticism in adults with ADHD: A qualitative study

**DOI:** 10.1371/journal.pone.0263366

**Published:** 2022-02-18

**Authors:** Danielle M. Beaton, Fuschia Sirois, Elizabeth Milne

**Affiliations:** Department of Psychology, The University of Sheffield, Sheffield, United Kingdom; Universitat d’Alacante, SPAIN

## Abstract

People with ADHD are at high risk of receiving criticism from others, yet criticism has not been well researched in this population. This study aimed to provide a rich understanding of what experiences adults with ADHD traits have with criticism. As part of a larger study, 162 participants with ADHD and high ADHD traits provided a written response to an open question asking about their experiences of criticism from other people. Thematic analysis was used to identify five common themes in the responses. Behaviours associated with inattention were perceived as the most criticised, whilst impulsive behaviours were mostly criticised in social contexts. Criticism was perceived via numerous conducts and was reported to have negative consequences for self-worth and wellbeing. To cope, some participants avoided criticism or changed how they reacted, including trying to accept themselves as they are. The responses indicated that receiving understanding from others played an important role in whether criticism was perceived. Overall, the findings highlight the need for more knowledge, understanding and acceptance towards neurodiversity from the general population.

## Introduction

Attention Deficit Hyperactivity Disorder (ADHD) is a prevalent, lifelong neurodevelopmental disorder. It is characterised by behaviours of inattention, impulsivity and hyperactivity that interfere with social and/or academic/occupational functioning [1]. In modern society these behaviours are not typically perceived as positive and so are often met with high levels of criticism from others [2,3]. Although criticism has been referenced in previous qualitative research on children and adults with ADHD [4,5], it has not been explored as a topic in its own right, so there is limited understanding of how criticism is experienced in those with ADHD.

Criticism is defined as negative evaluative feedback that occurs during social interactions [6]. Criticism can be person centred, whereby a person’s traits or abilities are evaluated, or it can be process centred, in which the processes or strategies used by a person to succeed are evaluated [6]. Similarly to praise, criticism can be a positive intervention to improve motivation and encourage others to adapt their behaviours to improve success [7]; however, whether it is positive or not is dependent on the delivery of criticism. Criticism directed towards the person rather than their strategies is more likely to impact levels of contingent self-worth and result in patterns of behaviour characterised by enhanced negative emotions, attempts to avoid future criticism and a likelihood to degrade ability and intelligence with negative self-cognitions [6,8]. Yet, it is this type of destructive criticism that is more likely to be perceived by people [9]. Perceived criticism reflects the amount of criticism that “gets through” [10]. High levels of perceived criticism are associated with the recurrence of depression and anxiety, and lower levels of self-esteem [10–12].

ADHD is one condition that is associated with high levels of receiving and perceiving criticism [13,14]. ADHD is highly heterogeneous, with different people displaying different combinations and severities of ADHD-related behaviours [15]. These symptoms are proposed to be a consequence of two broad domains of executive functioning (EF) which include inhibition and meta-cognition (working memory, planning/problem solving and emotional regulation) [16]. More specifically, hyperactive and impulsive behaviours are speculated to be a consequence of deficits in inhibition, whereas symptoms of inattention are a result of variations, in different domains of meta-cognition (16). Thus, in adults, symptoms of impulsivity may manifest through behaviours such as saying inappropriate things at inappropriate times, talking over others or being accident prone. Symptoms of hyperactivity can be observed through behaviours such as, talking excessively, fidgeting, and restlessness, whilst symptoms of inattention can be observed through difficulty focusing on and finishing a task, difficulties in planning tasks or keeping to time, making frequent mistakes, losing things frequently or seeming distracted [17].

Irrespective of whether a person with ADHD symptoms is diagnosed or undiagnosed, the persistent pattern of behaviours associated with the condition, are typically evaluated negatively. Children with ADHD are less well-liked than their neurotypical peers [18] and are more likely to be bullied during their school years [19]. In experimental studies, undergraduate students have reported lower levels of liking and reduced willingness to interact with people demonstrating ADHD behaviours [2,20]. Furthermore, parents are more likely to show higher levels of criticism and less warmth towards children with traits of ADHD [3]. Negative evaluations of people with ADHD are not dependent on their success or ability, as adults with ADHD who are considered high functioning still report higher levels of judgement from others [21]. It is therefore unclear what ADHD behaviours encourage these higher levels of criticism.

There are numerous qualitative studies that have attempted to capture the lived experiences of children and adolescents with ADHD, and recently these studies have been synthesised through meta-reviews [4,5] and thematic synthesis [22]. Across both of these systematic reviews, children and adolescents with ADHD frequently describe experiences of stigma, rejection or bullying, through name calling from peers, being scorned by teachers, active acts of bullying or being denied opportunities to engage in friendships and games [23–25]. Consequently, children and adolescents with ADHD describe feeling different [21,26]–like “square pegs” attempting to fit into “round holes” [27]. Although there is less qualitative data from adults living with ADHD, the few studies that have been conducted describe similar experiences to the reports of young people. In-depth interviews with older adults with ADHD and the qualitative analysis of personal narratives posted online reveal that many adults with ADHD report feeling misunderstood and believe that their experiences of criticism from parents and teachers have had consequential effects on their self-esteem [28,29]. McKeague et al., [28] found that the stigma and negativity directed towards people with ADHD resulted in stigma and negativity towards the self. Similarly, interviews conducted with four children with ADHD revealed that participants used the same descriptive words to describe themselves that others had used to describe them, indicating that self-appraisals may be built around others’ perceptions of them [30].

Thus, the qualitative research conducted to date has identified that criticism is a common theme across adults, adolescents and children with ADHD, and that criticism may have detrimental effects on self-perceptions [4,5,14]. Beyond that, our understanding of how people with ADHD experience criticism is limited. Accordingly, further research that expands this knowledge could have important implications for understanding the lived experiences of people with the condition and may highlight areas for intervention to reduce levels of criticism and improve the well-being of this population. The main aim of this study is to explore the experiences of criticism that adults with ADHD experience in more depth. To accomplish this, the current study aimed to find common themes in the experiences of criticism that people with ADHD perceive from their nearest family, friends and/or colleagues.

## Method

### Research design

The data used to conduct this qualitative analysis was obtained via a larger pre-registered study: https://osf.io/uwekq (13). The study was hosted online using Qualtrics (*www*.*qualtrics*.*com*) between January and March 2019. Participants were recruited via online ADHD forums, social media, university disability services and posters displayed publicly. Ethical clearance for the study was obtained from The Department of Psychology’s Research Ethics Committee at The University of Sheffield. Participants initially accessed the information form and consent sheet via an online link to the study. In the study, participants completed a number of self-report questionnaires (see Beaton et al. [13] for more information) and were also offered the opportunity to respond to an open question that asked participants *“…to share [their] experiences of criticism from the people in [their] nearest environment (e*.*g*. *family*, *friends*, *colleagues*‡*)*.*”* An open text question was used to collect the data because participants could respond anonymously without any researcher input. Participation in the study was completely anonymous; therefore, after participants submitted their responses we could not identify, nor contact participants to give feedback about the study or request follow-up information. This was to ensure that the responses would be free of any potential sampling, procedural, response, or interviewer bias that occurs with face-to-face qualitative research with a smaller group of participants.

As the study aimed to explore the experiences of people with ADHD, participants were screened for ADHD symptoms using The Adult ADHD Self-report Scale V1.1 (ASRS-V1.1) [31]. The ASRS asks participants to rate the frequency of ADHD symptoms on a scale ranging from 0 (never) to 4 (very often) on six items that reflect the clinical criteria in the DSM-IV (APA 2013). It is recommended that individuals who report “sometimes”, “often” or “very often” to the first three questions, or “often”/ “very often” to the final three questions more than 4 times in the questionnaire, have symptoms highly consistent with ADHD. This scale therefore provides a categorical classification of ADHD and identifies people with high levels of ADHD traits. We wanted to ensure that the experiences reported in this study were fully inclusive and did not discriminate based on access to clinical intervention, which can depend on location and wealth. Consequently, for this study, participants whose ASRS responses identified them as highly consistent with ADHD as defined above, were included in our sample. To be fully transparent, participants’ diagnostic status is reported alongside any quotes referenced below: ADHD+ reflects participants who self-reported as having a diagnosis of ADHD, and ADHD- identifies participants who self-reported that they did not have a diagnosis of ADHD but reached the cut-off for high ADHD traits on the ASRS. ADHD often has a high rate of co-occurrence with other conditions, such as mood disorders (e.g. depression, anxiety, bipolar, Obsessive Compulsive Disorder [OCD]), personality disorders (e.g. Borderline Personality Disorder), Autism Spectrum Condition (ASC), and other behavioural conditions (e.g. conduct disorder) [17,32,33]. However, we wanted to ensure that the experiences captured in this study were representative of responses towards ADHD specifically, and not a potential consequence of negative cognitive biases associated with mood disorders [34], or stigma towards symptoms of other conditions [35,36]. Therefore, to ensure the experiences reported were not a consequence of another condition, the responses from participants that self-reported a co-occurring condition were removed from the dataset. Finally, to ensure that the data included meaningful information, responses with less than 10 words were not included in the analysis.

In total 498 participants high in ADHD traits provided a free text response to the question. Of these, 298 responses were removed because participants reported at least one co-occurring disorder (ASD = 10, OCD = 2, Mood Disorder = 187, Behavioural Disorder = 1, Other = 22, multiple conditions = 76), a further 44 responses were removed because they had fewer than 10 words. Thus, the final sample consisted of responses from 162 individuals: 109 females, 52 males and 1 person who identified as other. Participants’ ages ranged between 18–62 (*M* = 33.80, *SD =* 10.60). In total 96 participants self-reported that they had a clinical diagnosis of ADHD with an average ASRS score of 18.97 (*SD* = 2.70), while 66 participants did not report a diagnosis of ADHD but their ASRS scores indicated high traits consistent with ADHD (*M* = 17.70, *SD =* 3.16). The severity of ADHD traits was significantly greater in those who self-reported an ADHD diagnosis (*t* (160) = -2.75, *p* < .05). The full demographic information of the sample is presented in Table 1.

**Table 1:** The demographic details of the sample.

	N (162)
**Place of Residence**	
United Kingdom and Ireland	109
USA and Canada	34
Europe	10
Australia/New Zealand	3
**Ethnicity**	
Caucasian	141
Mixed Ethnicity	8
Other	7
**Employment**	
In employment	123
Unemployed	35
Disabled/Sickness Leave	4
**Highest Education Level**	
Secondary school:	18
College/Sixth form:	35
University degree:	47
Postgraduate / Professional Degree:	42
**Relationship Status**	
Single	44
In a relationship/ Married / Co-habiting	107
Separated / Divorced	11

### Researcher description

The data was analysed by the first author DMB. DMB is a white female, with personal and professional experiences with ADHD. She has engaged with adults and children with ADHD as a researcher, but also through clinical settings offering legal and psychological support. Prior understanding of what experiences adults with ADHD may perceive, arise from personal observations that DMB made in these settings. It is these observations which incentivised the inclusion of an open-ended question in the study. DMB was also aware that participants with ADHD and high traits of ADHD in this same sample had reported that they perceived higher levels of criticism from others from the quantitative analysis as part of the wider study (Beaton et al., 2020).

### Data-analytic strategies

Data exclusion and preparation was conducted in Microsoft Excel, whilst analysis was conducted in NVivo-12, a software designed for qualitative analysis to allow for intuitive coding of data. Thematic analysis was conducted by DMB using the methods outlined by Braun & Clarke [37], in order to identify common experiences of criticism. An iterative process was used over 5 phases of analysis using inductive coding, thus coding categories emerged from the data throughout analysis. During phases 1–2, DMB familiarised herself with the data, and began coding the data semantically. During phase 3, patterns in the codes were considered, and possible themes were identified. In phases 4–5 the cycle of analysis was repeated to review and refine the themes and the second and third authors reviewed the results, to ensure that the codes work well in relation to the research question, the datasets and the codes. The codebook that was developed is presented as a table in S1 Table.

## Results

Five themes, divided into multiple sub-themes, were identified in the text. The themes and sub-themes are presented in Table 2. The responses of 11 participants did not contribute to any of the themes identified. Participant number (e.g. P1) and ADHD diagnosis are presented alongside quotes. ADHD+ represents participants who self-reported an ADHD diagnosis, ADHD- represents participants who did not self-report an ADHD diagnosis, but who met the criteria on the ASRS-V1.

**Table 2:** Themes identified, sub-themes, how many references correspond to each sub-theme, and an example of each sub-theme.

Theme	Sub-theme	Number of quotes (% of theme)	Percentage of total statements	Example (Participant number, ADHD diagnosis +/-)
**1.** What is criticised?		93	32%	
	“Everything I do”	6 (6%)	2%	*“My husband seems to be unhappy with whatever I do*.*” (P*.*13 ADHD-)*.
	Organisation	18 (19%)	6%	*“Family and occasionally friends tell me how disorganised and messy I am*.*” (P*.*149*, *ADHD+)*.
	Time management	13 (14%)	4%	*“I am often criticised for being late…” (P*.*22*, *ADHD-)*
	Impulsivity and self-control	23 (25%)	8%	“*I have been told many times that I am over reacting*, *that I am causing discussion*, *I can’t control myself…” (P*.*38*, *ADHD-)*
	Forgetfulness	14 (15%)	5%	*“Frequent comments*, *jokes and sarcasm about my level of forgetfulness which are unintentionally hurtful*.*” (P*.*29*, *ADHD-)*.
	Focus and inattention	19 (20%)	7%	*“My mom likes to point out when I’m daydreaming about the future” (P*.*112*, *ADHD+)*
**2.** What is perceived as criticism?		62	21%	
	Comparisons with others	8 (13%)	3%	*“Criticism from parents for not being the same as everyone else…” (P*.*143*, *ADHD+)*
	(Mis)judgement	13 (21%)	4%	*“I feel like a lot of my family and acquaintances think I’m pitiful or stupid/lazy*.*” (P*.*42*, *ADHD-)*
	Expectations	11 (18%)	4%	*“I always feel pushed and pulled in so many different directions trying to please everyone*.*” (P*.*44*, *ADHD-)*
	Humour	7 (11%)	2%	*“My late dad upon learning my diagnosis just mocked me and never let me forget my mistakes” (P*.*99*, *ADHD+)*.
	Others emotional reactions	10 (16%)	3%	*“My family is extremely critical—if I make a mistake I either get yelled at or mocked for it (and I’m almost 40 and don’t live with them*!*)” (P*.*121*, *ADHD+)*.
	Rejection	12 (19%)	4%	*“I have had a socially traumatic experience where people who I thought I was close to deliberately alienated me without a given reason” (P*.*100*, *ADHD+)*.
**3.** Consequences of criticism		53	18%	
	Sensitivity to criticism	27 (51%)	9%	*“Criticism wounds me deeply even if it is not intended*. *I feel quite brittle” (P*.*56*, *ADHD-)*
	Altered self-perceptions	26 (40%)	9%	*“I don’t have much respect for me*.*” (P*.*61*, *ADHD-)*
**4.** Coping with criticism		45	15%	
	Hiding ADHD	12 (27%)	4%	*“Colleagues don’t really criticize me much*, *I work very hard to have good performance overall and keep that from happening*.*” (P*.*81*, *ADHD+)*.
	Active change	7 (16%)	2%	*“I’ve surrounded myself with supportive people who provide criticism in order to help me realize my flaws and help me improve myself” (P*.*67*, *ADHD+)*
	It’s not me it’s them	6 (13%)	2%	*“I believe the cause for this trouble is their own undiagnosed mental health issues*.*” (P*.*107*, *ADHD+)*
	Openness to criticism	10 (22%)	3%	*“I’m aware that people have their criticisms of me from time to time*, *but it’s generally only a sign that they want to be helpful or don’t understand the nature of my struggle*.*” (P*.*136*, *ADHD+)*
	Knowing and accepting the self	10 (22%)	3%	*“I know my flaws and can ignore those who don’t understand or accept me*.*” (P22*, *ADHD-)*
**5.** The role of support and understanding		38	13%	
	Why do others lack understanding?	4 (11%)	1%	*“I am met with very little understanding from my family*. *I am doing what needs to be done and working hard…” (P*.*41*, *ADHD-)*
	Misunderstanding as a precursor to criticism	16 (42%)	5%	*“I think that some of my friends don’t understand to the extent I am unable to do things without being medicated*, *and that I can’t simply force myself into a schedule and magically fix everything*, *even getting out of bed and getting dressed requires focus I am sometimes unable to summon*.*” (P*.*106*, *ADHD+)*
	The benefits of understanding	18 (47%)	6%	*“My boyfriend is incredibly understanding and supportive of me because he understands my ADHD and how it effects[sic] my behaviours*.*” (P*.*88*, *ADHD+)*.

### What is criticised?

This theme captured the most commonly criticised behaviours and traits that participants reported. It was the most commonly discussed topic, with 93 (32%) statements contributing to this theme. A number of participants responded to the question by listing traits/behaviours that are frequently criticised:

“*forgetful*, *aloof*, *mindless*, *late for everything*, *doesn’t respond to emails*, *misses deadlines” (P*.*94*, *ADHD+)*

#### “Everything I do”

Many participants with high traits of ADHD portrayed the criticism they received as comprehensive, rendering that they were consistently criticised and felt unable to succeed:

“*I never seem to be abke [sic] to satisfy anyone*. *Always fail*, *especially at basic family life*.*”* (P.31, ADHD-).

#### Organisation

Having a lack of organisation, or skills to be organised was a commonly reported factor that was highly criticised by others. Some participants reported this directly:

“*Colleagues say I’m disorganised…” (P*.*55*, *ADHD-)*.

Other participants reported criticism towards organisational skills through behaviours that represent planning and organisation:

“*He criticizes my inability to finish tasks*, *start in a timely manner*, *and maintain hobbies or outside relationships*.*” (P*.*166*, *ADHD-)*

#### Time management

Another behaviour that was commonly met with negativity was time keeping. Many participants referenced criticism regarding their limited awareness of time and their inability to be on time to appointments and meetings:

“*My mother will often get disproportionately angry with me for not doing something in a timely manner*, *usually implying that it is an easy task and if I really cared about getting it done I would*.*” (P114*, *ADHD+)*

#### Impulsivity and self-control

Many participants reported that they were criticised for a lack of self-control and for engaging in more impulsive behaviours:

“*Im [sic] told Im [sic] unfocused and unfiltered*. *Im [sic] constantly criticized for being neurodiverse*.*” (P*.*93*, *ADHD+)*.

This was most often reported through the behaviours that people engaged in. A lack of self-control in social situations was the most frequently reported behaviour, with patterns of interrupting others, saying unnecessary things, or talking too much or too loudly:

“*At work people often criticise me on how much I talk and tell me to take a breather*.*” (P*.*124*, *ADHD+)*

Many participants also discussed how their emotional impulsiveness was a target for criticism:

*“The people close to me would say I’m quick to anger…” (P.57*, *ADHD-)*

“*Criticism from parents … for having emotions and letting them affect me” (P*.*143*, *ADHD+)*

#### Forgetfulness

Many participants reported that they have been, or are criticised for their poor memory:

“*I’ve been criticised for my forgetfulness*…*just write things down like we do*!*” (P*.*92*, *ADHD)*

#### Focus and inattention

Another behaviour that was frequently reported as a target of criticism was the persons abilities to pay attention and focus:

“*I am just the strange one or the clever one who let them down because I didn’t focus enough” (P*.*91*, *ADHD+)*

“*In work people can be critical when I’m easily distracted…” (P*.*132*, *ADHD+)*

People also reported criticism towards inattention through descriptions of the person using words like *“…mindless” (P*.*94*, *ADHD+)*, *“spacey” (P*.*126*, *ADHD+) and “…ditzy…” (P*.*17*, *ADHD-)* and about the persons levels of clumsiness or carelessness:

“*I am also often criticised for being clumsy*.*” (P*.*22*, *ADHD-)*

“*Careless mistakes always pointed out even when not a huge issue*.*” (P*.*73*, *ADHD+)*

### What is perceived as criticism?

The criticism that participants reported was not always perceived through direct comments about the person’s behaviour as was seen in Theme 1. In total, 62 (21%) statements referenced indirect methods that were perceived as forms of criticism, such as: making comparisons between them and other people, judgement from others, meeting expectations, “….mak[ing] fun…” (P.82, ADHD+), and through acts of rejection.

#### Comparisons with peers

Many participants reported that they felt criticised when they were compared to other people. One participant reported that they were compared directly to others in regard to successes and failures:

“*My parents tend to be criticize [sic] me and my career decisions by comparing me to others my age who are relatively more successful than me*.*” (P*.*52*, *ADHD-)*.

Whereas others recounted comments that had been made to insinuate the person is different from others:

“*…why can’t you be like everyone else…” (P*.*80*, *ADHD+)*

#### (Mis)judgement

Criticism through a sense of disapproval from others was reported through the judgements that others put upon them. This was reported as a general sense of judgement or through judgement of specific behaviours:

“*I feel I’m being judged at work…” (P*.*89*, *ADHD+)*

One participant commented that they *“…often feel misjudged by people close to me…”* (P.51, ADHD-). These potential misjudgements can be seen through the adjectives that participants reported to criticise them, providing a misjudgement of character as *“…messy…” (P*.*149*, *ADHD+; P*.*17*, *P*.*44*, *ADHD-)*, *“…too flaky…” (P*.*78*, *ADHD+)* or *“… lazy…” (P*.*96*, *ADHD+*, *P*.*42*, *P*.*45*, *ADHD-)*.

#### Expectations

Some participants perceived the expectations that others had of them as a form of criticism. These expectations were reported as general or on specific behaviours:

“*Mostly family as they have high expectations*. *My friends expect me to be more organised and on time than I am most of the time*.*” (P*.*153*, *ADHD+)*.

As a result, some participants felt that they frequently *“…let people down*.*”* (*P*.*59*, *ADHD-)*. One interpretation is that it is the inability to meet the expectations, that makes the expectations feel critical.

#### Humour

Another way that subjects reported criticism was through the use of humour. Many participants reported that their behaviours or diagnosis were used as the basis of jokes:

“*My family often teases and makes fun of me for my forgetfulness and restlessness*, *as well as for being clumsy and inattentive” (P*.*82*, *ADHD+)*.

Bringing attention to negative behaviours or traits in the person, and laughing at them was reported as hurtful:

“*Frequent comments*, *jokes and sarcasm about my level of forgetfulness which are unintentionally hurtful” (P*.*29*, *ADHD-)*.

#### Others emotional responses

Perceiving others negative emotional reactions was also considered as criticism for some participants. Many participants reported negative emotions such as frustration, annoyance and anger directed towards them. One participant reported:

“*My family is extremely critical—if I make a mistake I either get yelled at or mocked for it (and I’m almost 40 and don’t live with them*!*)” (P*.*81*, *ADHD+)*.

#### Rejection

The final subtheme consisted of criticism through acts of rejection. Participants reported that they felt as if they were *“…treated less favourable…” (P*.*77*, *ADHD+)* and *“not accepted socially” (P*.*120*, *ADHD+)*. Other participants reported more specific experiences of being rejected, as illustrated in the following quote:

“*I would present ideas ect which were ignored or given to someone else*. *I would say things be ignored and then someone would say the same thing and they would love it*!*” (P*.*96*, *ADHD+)*.

#### The consequences of criticism

This theme captures the various outcomes that participants reported as a result of the criticism. In total, 53 (18%) statements contributed to this theme. Some participants reported their immediate responses to criticism, whereas others discussed the longer-term consequences that criticism has had on themselves and their behaviours.

#### Sensitivity to criticism

Many people disclosed their emotional reactions to criticism and how it made them feel in the short term and long term. Although it is somewhat expected that people would have a negative response to criticism, many participants reported that they had particularly strong reactions to criticism:

“*Any suggestion*, *critique or something similar from anyone cuts like a knife and leaves me unable to feel anything but devastated for days*.*” (P*.*129*, *ADHD+)*.

A greater sensitivity to criticism was also perceived through participants enhanced awareness of criticism. Participants reported sensing or perceiving criticism from others without any direct evidence of critical evaluation:

“*For the most part criticism has not been brought up to me in my new job but I can not [sic] shake the feeling the [sic] are highly critical of me*. *Even though I have no proof of it” (P*.*127*, *ADHD+)*.

Multiple participants also reported that they believed others were critical of them privately by thinking poorly of them or being critical of them behind their backs:

“*I can’t actually think of any specific experiences*. *When I look at it logically I know that my family and friends aren’t hugely critical of me*, *but I get very worried that people are critical of me behind my back*, *and…I’m convinced that people are criticising me*.*” (P*.*144*, *ADHD+)*

For one participant, the sensitivity to criticism consumed them, resulting in the person overgeneralising critical evaluation to neutral comments:

“*I listen to all feedback and take everything to heart*, *even if not directly aimed at me*. *I’m consumed with the feeling I’m not good enough*. *An example is when my husband moans about the messy pan cupboard it makes me feel like a failure*. *I know it’s not exactly directed at me but I take it to heart and it makes me feel inadequate and like I can’t get anything right*. *This is true for most things*.*” (P*.*44*, *ADHD-)*.

#### Altered self-perceptions

The criticism received by others also had detrimental effects on many of the participants’ sense of self. Some participants reported that criticism had led them to feel that they had little value:

“*…go through life feeling worthless because no one should feel that way about themselves but after years of criticism and being made to feel like it was all in your head the damage has already been done*.*” (P*.*91*, *ADHD+)*.

The resulting low sense of worth was also observable through the sense of being a burden to others:

“*I don’t have many friends and feel a burden to the ones I have which makes me think they are not really my friend*.*” (P*.*8*, *ADHD-)*.

In addition, many participants reported how criticism had impacted their self-esteem and confidence in themselves and their abilities:

“*They have caused a lot of pain and self-esteem issues*.*” (P*.*107*, *ADHD+)*.

For one participant, the criticism from others impacted their sense-of-self to a point where the criticism was internalised and resulted in them also being critical towards themselves:

“*My mother is extremely critical and obsessive and as I live with her*, *it’s hard to separate myself from negative criticisms which fuel my own negative opinions of myself*.*” (P*.*103*, *ADHD+)*.

For other participants, they felt as though they had to change themselves and their behaviours in order to reduce the criticism:

“*Always told I talk to [sic] much makes me feel I constantly have to try hard not too” (P*.*20*, *ADHD)*.

### Coping with criticism

This theme covers the various behaviours that participants reported to cope with criticism and/or to protect themselves from criticism. In total, 45 (15%) of the total statements contributed to this theme. The approaches varied between people, with some participants engaging in more insular methods, such as becoming more open to criticism, being more aware/accepting of the self, or viewing the criticism as indicative of other people. Others reported externalised behaviours to avoid criticism, such as taking control of relationships, and changing their environment and social interactions in order to avoid criticism.

#### Hiding ADHD

Many participants reported making an effort to hide their ADHD diagnosis and/or symptoms in order to avoid criticism. Some participants who self-reported as having an ADHD diagnosis stated that they did not inform people about their ADHD diagnosis:

“*I have yet to tell my parents about my adhd diagnosis for example*. *But then by hiding things it also causes me great anxiety*. *Catch 22 situation*.*” (P*.*74*, *ADHD+)*

One participant reported that they hide their ADHD because they had previous experiences of rejection after their diagnosis had been disclosed:

“*Once employment knows I have Adhd*, *they either don’t employ me or look for ways to fire me*. *Thus I’ve learnt to hide it*, *well*.*” (P*.*150*, *ADHD+)*.

A variety of different methods were also used by participants to actively hide their ADHD symptoms. One reported strategy was to lower their ambitions and work in settings where others had lower expectations of them so that their ADHD symptoms cannot cause them to fail and consequently receive critical evaluation:

“*These days I do not get much criticism from those around me*, *but I believe that’s because I’ve consciously gotten myself to a place where expectations are low*. *I’m in a job that is frankly beneath me*, *because I don’t trust myself to succeed at something that actually challenges my abilities*.*” (P*.*104*, *ADHD+)*.

Another common strategy used by many participants was to increase the time and effort put into work. Participants reported that they adopted a “*…hardworking ethos…”* in order to “…*combat for feeling inadequate on the job…” (P*.*99*, *ADHD+)*. It was disclosed that this strategy could be at the detriment of their relationships and wellbeing:

“*40 years of what was deemed to be failing really took it’s [sic] toll on my wellbeing*. *Mainly due to lack of self care as I was using everything I had to prove that I can*. *There is no differentiated learning” (P*.*90*, *ADHD+)*

Some participants actively attempted to avoid criticism by socially withdrawing themselves:

“*I avoid conversations where I might have to explain myself*.*” (P*.*74*, *ADHD+)*.

Hiding symptoms of ADHD was said to cause *“…great anxiety” (P*.*74*, *ADHD+)* and to take a lot of effort:

“*The exhaustion of fear that people will realise how much you ’cover’ your forgetfulness with little white lies and the looks of suspicion and/or disapproval if suspected” (P*.*29*, *ADHD-)*.

#### Changing the situation

A second branch of strategies to protect the self from criticism involved participants changing their circumstances. If participants were not naturally surrounded by understanding, accepting or supportive people, they described actively changing their surroundings to ensure that they were:

“*I have no problem cutting off those that don’t have good will towards me*, *even if they are family members or old time friends*. *Life is hard enough without having to put up with unjust behaviour” (P*.*131*, *ADHD+)*.

#### It’s not me, it’s them

Some participants protected their wellbeing and sense-of-self by viewing criticism as more indicative of the person being critical, rather than a reflection of who they are. Some people attributed the criticism as a consequence of the other persons mental health:

“*…she clearly has issues of her own because she tells me that my missing things means I don’t value what she values and I don’t care about the things she cares about*, *or I don’t care that she cares about them and am deliberately trying to thwart her*.*” (P*.*136*, *ADHD+)*.

Whilst others viewed criticism as an opinion, rather than an accurate judgement:

“*I have become used to receiving criticism for various reasons*. *I am able to accept it is part of having an opinion but not to take too much personally*.*” (P*.*11*, *ADHD-)*.

#### Openness to criticism

Some participants described their acceptance of criticism and perceived it as an opportunity to ignite self-improvement. An openness to criticism in many participants was associated with a more positive perspective of the critical evaluations and of other people’s motivations:

*“I am happy to receive positive criticisms from friends and family. I am close to my friends and partner and I trust they are tolerant of me and have my best interests in mind. Their advice has been helpful to me in my life.” (P.100, ADHD+)*.

Nevertheless, even if the criticism is well received, it can still have negative emotional consequences:

“*I am often forgetful and spacey*. *Sometimes*, *I don’t think things through*. *These criticisms are fair and I always keep it in mind*, *however it still causes me to have anxiety sometimes*.*” (P*.*126*, *ADHD+)*.

#### Knowing and accepting the self

Many participants discussed how their levels of self-acceptance and/or self-knowledge contributed to how they perceived and/or reacted to criticism. They reported that they had engaged in an active efforts to learn more about themselves and to understand their personal ways of reacting to the world:

“*In the past I would feel very misunderstood and criticised but learning more about myself and my ADHD has really helped me to put things into perspective and I am less inclined towards paranoia*, *and more inclined to hold back from reading into every little thing*.*” (P*.*69*, *ADHD+)*.

### The role of support & understanding

A number of statements (38, 13%) also described how understanding and support was associated with criticism. It was expressed in two ways; the first described criticism in relation to a lack of understanding, whilst the second discussed levels of criticism in relation to the presence of support and understanding.

#### Why do others lack understanding?

Participants with diagnosed ADHD stated that their condition is often dismissed by people:

“*My parents don’t believe in Adhd etc because they know no better I am just the strange one or the clever one who let them down because I didn’t focus enough*.*” (P*.*91*, *ADHD+)*

Some participants believed that it was this refusal to acknowledge ADHD as a condition that prevented others from understanding the challenges and difficulties that people with ADHD experience:

“*Whilst I’m very open about my condition*, *my experience is that even people close to me don’t take account of it and still react and interact with me on [sic] with little regard to it*. *It’s very much a condition that refuses to be recognised*, *even by people who are well aware of it*.*” (P*.*121*, *ADHD+)*.

In contrast, one participant discussed that receiving a diagnosis changed how others were critical towards them:

“*Parents and previous relationships would say I did it on purpose now it’s accepted as part of me and not deliberate” (P*.*85*, *ADHD+)*

#### The role of misunderstanding

Participants’ responses inferred that a lack of understanding may lead to the criticisms that others have of them. Many participants discussed how a lack of understanding from others contributed to the (mis)judgements that were made of them which resulted in a sense of being *“…looked down on” (P*.*107*, *ADHD+)*:

“*My husband is sympathetic but doesn’t really understand ADHD*. *So quite often he’ll be exasperated with something I’ve not done or how I’ve reacted to something*. *And he’ll ask in frustration “what’s wrong with you*?!,*" "Why are you reacting like that*?!*" etc*. *And it’ll be my ADHD symptoms*. *I feel like he doesn’t care enough to understand my struggles and to help me with situations I find challenging*.*” (P*.*79*, *ADHD+)*.

#### The role of understanding

It seemed important to many participants that although others close to them may be critical of them, they were also understanding:

“*My parents are critical of me but understand” (P*.*88*, *ADHD+)*

Multiple participants reported that they were “…lucky…” *(P*.*34*, *ADHD-)* and “…fortunate…” *(P*.*64*, *ADHD-)* to have supportive and understanding people around them, and levels of understanding and support were often described as an opposing feature to criticism.

“*My friends are extremely supportive and not critical at all*.*” (P*.*103*, *ADHD+)*

Some participants discussed how others understanding limits the criticism that they give, but is more likely to be associated with more supportive behaviours:

“*Prior to diagnosis and treatment*, *I would struggle to share the chores with my partner… During arguments*, *he would suggest I was just lazy and irresponsible*. *These were thoughts I always had about myself*. *The criticism has vanished entirely now that we both understand why I struggle…”(P*.*161*, *ADHD+)*

## Discussion

The main aim of this study was to attain a rich understanding of how criticism is experienced in adults with ADHD. Conducting a thematic analysis of open text responses to the question, “*…share your experiences of criticism from the people in your nearest environment”*, enabled us to attain an insight into the criticism associated with ADHD traits and its impact. Our findings highlight what behaviours are most commonly criticised in people with ADHD traits, what people with ADHD traits perceive as criticism, what consequences arise from criticism, how people with ADHD traits cope with the criticism, and the important role that understanding plays towards the presence of criticism.

The first theme identified what traits participants felt were criticised, which has not been revealed in previous research but has implications for social, educational and personal intervention. Importantly, there was a high degree of overlap in what participants felt they were criticised for, which suggests that particular ADHD behaviours are perceived negatively, and warrant judgement from others. Some participants perceived criticism toward numerous qualities or felt that they felt they were criticised for ‘everything’, but the majority of participants reported criticism towards specific traits and behaviours. This provided an insight into what symptoms of ADHD may be most vulnerable to the negative evaluation of others. The behaviours and qualities reported are characteristic of the differences in executive functioning that are theorised to underpin ADHD. In particular, behaviours that are theorised to embody differences in meta-cognition, and therefore inattention, were reported as the most criticised. Reports of criticism towards behaviours of organisation, focus, forgetfulness and time management made up 90% of the behaviours that were reported in the first theme. This could suggest that behaviours of inattention are the symptoms most negatively evaluated by others, or that criticism towards these behaviours are perceived with a greater sensitivity by those with ADHD traits. It is possible that difficulties in planning, organisation, memory, and time management are not well-known consequences of inattention to neurotypical others, and therefore are misjudged as a consequence. This is supported by the critical adjectives that were reported to describe the participants, such as: “unfocused”, “careless”, “forgetful”, “lazy”, “disorganised” and “messy”. Consequently, this may indicate that improved education around what characteristics arise as a consequence of ADHD, could reduce the level of criticism towards those with high traits of the condition.

Another important pattern identified was that the majority of the critical characteristics represented difficulties in social interaction, including interrupting others, being too loud/quiet or saying inappropriate things. Previous research has found that adults with ADHD feel that they struggle in social situations for similar reasons as reported here [26]. However, the responses from participants in this study indicated that it is the feedback they perceived from others that made them feel that they are behaving in a non-typical fashion. Misunderstanding around these behaviours were described by some participants as a catalyst to ending relationships–a finding similar to that reported by Nyström et al. [26]. This is also in line with evidence that adults and children with ADHD are more likely to be socially rejected by their peers [18]; therefore, it is possible that these criticised behaviours contribute to the social rejection that people with ADHD are vulnerable to. However, it is still unclear why these behaviours in particular are negatively evaluated by others. Future research could consider investigating what neuro-typical individuals’ perspectives are of these behaviours in order to understand why they may warrant criticism and potential rejection.

Prior to this study, little was known about what adults with ADHD perceive as criticism. However, the second theme of this study clarified that criticism is perceived via multiple means, which may have implications for the self-worth of those with ADHD. Burhans & Dweck’s [38] contingent worth model suggests that people document self-worth via the judgements and evaluations of others to meet certain standards, so any behaviours that provide evidence of negative evaluation could be seen as a threat towards, or confirmation of, an individual’s contingent worth. The second theme identified that criticism was perceived through jokes, comparisons with others, judgement, rejection and other peoples’ expectations. This highlights that criticism was not just perceived through verbal reproach, but that people with ADHD traits are sensitive to exchanges that may not be intended as criticism. Over time, this could contribute towards the reduced levels of self-worth that participants reported in the study, and in other studies [5,39,40]. What is more, people with ADHD respond with heightened sensitivities to reward and punishments [41]. Consequently, parenting interventions such as the well-known Triple-P positive parenting program [42] advocate that negative behaviours should be ignored, and positive behaviours praised. The results of this second theme could have important implications for the efficiency of such behavioural interventions because the results show that criticism is perceived where punishment may not be intended. Thus, it may be important for parents of children with ADHD to ask the child what they perceive as criticism. This also extends into adulthood to maintain good romantic relationships, friendships and work relationships. Overall, this theme suggests that family members, friends and employers or teachers of people with ADHD should be particularly attuned to how they communicate their frustrations or annoyances towards people with ADHD and adjust accordingly.

The third theme identified the effects that criticism had on the participants. Firstly, the findings revealed that participants felt they had lower levels of self-worth as a result of the criticism. Thus, this current study adds to a body of research that suggests that the criticism from others may lead to low self-esteem [43], increased self-criticism [44], and heightened feelings of self-shame [26], all of which have important implications for the development of self-concept, self-esteem and mental health. Secondly, the emotional responses to the criticism were reported to be more intense than expected and many participants reported a heightened awareness and paranoia of criticism, judgement or rejection. This is in line with many ADHD information websites that refer to a phenomenon called ‘rejection sensitivity dysphoria’, which suggests that people with ADHD have more severe emotional responses to criticism and rejection than others [45]. When neurotypical people experience recurring negative feedback they are more likely to develop an oversensitivity towards the criticism [46]. Accordingly, it is possible that adults with high traits of ADHD are more sensitive to criticism because they experience it more frequently. However, being more sensitive to criticism can have detrimental effects on wellbeing and mental health [47,48]. Moderating the criticism towards individuals with ADHD may be important to protect the mental health of this population.

Despite research suggesting that people with ADHD experience higher levels of criticism compared to others [2,3], this is the first study we are aware of to provide insight into how people with ADHD cope with that criticism. Some participants coped with criticism by trying to observe it as a more positive concept and not seeing it as a true representation of them. Those who have a more adaptive, mastery-oriented, response to criticism are more likely to perceive criticism as a helpful aid, which is associated with reduced negative affect in response to the criticism and an increased persistence and motivation to succeed through constructive changes [6,38]. Many participants used self-awareness and self-acceptance as a way to alter how they perceived and responded to criticism to this more positive and potentially healthy pattern of behaviours, however others coped with criticism by avoiding situations that could evoke criticism and/or people who were more likely to be critical. ADHD is highly stigmatised and people can respond negatively to the label of ‘ADHD’ before even observing any ADHD symptoms [2,49,50]. This provides a rationale as to why participants in this study and in previous interviews [51] reported that they attempted to hide parts of themselves to prevent others’ judgement and criticism. One interpretation of this behaviour is that some people with ADHD believe the criticism is indicative of something ‘wrong’ with them that they need to eliminate in themselves. The findings also indicated that this has costs associated with it, through reduced wellbeing, inhibited academic, occupational or personal achievement and overexertion of energy, therefore it could be argued that this is not a positive way of coping with criticism. In contrast, other participants avoided criticism by surrounding themselves with people who were supportive and understanding and rejecting people who were more critical of them. One interpretation of this behaviour is that some people with ADHD believe the criticism is not indicative of something ‘wrong’ with them, but they just need others around them who are more accepting and supportive of diversity. It could be argued that this method of avoidance is a more positive way of coping with criticism.

The importance of understanding and acceptance towards ADHD was a prominent theme across participants’ responses, with some participants discussing the positives of others understanding, and others describing the negatives when others do not understand. From participants’ responses, it was found that not understanding *why* certain behaviours may be present appeared to result in criticism or judgement that the respondents felt were misrepresentative of their character and ability. Feeling misunderstood and misjudged due to ADHD behaviours has been reported previously [29], emphasising that people in the general population may not understand how ADHD presents itself. On the other hand, some respondents defended critical behaviour when they felt that others were also understanding, which may be an indication that understanding mitigates or outweighs criticism. This proposal is consistent with research examining adolescents with ADHD who stated that they inform others of their ADHD diagnosis so that if they were criticised, the other person would understand why they do that behaviour [4]. Overall, the results of the current study suggest that understanding the behaviours of someone with ADHD encourages more supportive behaviour in place of criticism. This conclusion has been drawn from multiple other qualitative studies interviewing people with ADHD [28,30,52], thus, this study contributes to the popular theory that improved understanding of ADHD will ultimately change the negative perceptions that people have of the condition, and may subsequently alter the level of stigma towards them [49,50,53]. This is a particularly important consideration for adults with ADHD who report that positive, accepting and supportive relationships with others contributes to improved self-esteem and wellbeing [29].

### Strengths and limitations

On the whole, using an open text question has allowed for an unbiased and comprehensive insight into the incidents of criticism that adults with ADHD frequently experience. A particular strength of the study is that the responses were unguided and completely anonymous, which increases confidence that the results are free of any potential sampling, procedural, response, or interviewer bias. Moreover, by using this method we have also provided an insight into what people with ADHD traits want to discuss around the topic of criticism. Nevertheless, being unable to ask follow-up questions and prompt further discussion does mean that the responses we received were limited. Being able to interact with the participants face to face may have led to answers with more depth and clarity.

Although eliminating people with co-occurring conditions adds reassurance to the findings being attributed to the traits of ADHD, participants were not screened for co-occurring conditions and we replied on self-report. Therefore, some participants in the sample may have chosen not to disclose that information. In contrast, removing participants with co-occurring conditions could also be considered a potential shortcoming of the study. ADHD is a highly comorbid condition [17,32,33], therefore the results of this study may not be representative of the wider population of ADHD that also experience symptoms of another condition. Moreover, participants’ diagnostic status was not clarified by a clinical professional. Although all participants met the clinical threshold on the ADHD screening tool, relying solely on self-reported diagnosis may lack external validity, and may have resulted in an overinclusion of participants which would not be diagnosed with ADHD by a clinician.

## Conclusions

Overall, this study has provided an in-depth understanding of the experiences that people with ADHD traits have of criticism. The findings highlight the need to consider what individuals with ADHD perceive as criticism, how they respond to criticism, and what potential effects this may have on their wellbeing. The results also identify several gaps in the literature and directions for future research, including: how neurotypical people perceive neuro-diverse individuals; if people with ADHD are inherently reactive to criticism or if sensitivity to criticism is a consequence of environmental factors; and, whether changing how people with ADHD respond to criticism improves levels of wellbeing and/or educational and occupational outcomes. Importantly, the study demonstrates that levels of understanding are a fundamental factor in the negative evaluations that others have of people with ADHD. In turn, the results suggest that improving understanding may lead to more positive and supportive relationships, and that the level of knowledge and understanding that neuro-typical people have of ADHD may help to reduce criticism towards those with the condition. The current findings also highlight the importance of advocating for a more flexible society that is accepting of individuality and neurodiversity.

## Supporting information

S1 Table The codebook developed for the thematic analysis. (PDF)
