# Supplementary material for: Experiences of criticism in adults with ADHD: A qualitative study
Source: PLoS One. 2022 Feb 18;17(2):e0263366. doi: 10.1371/journal.pone.0263366 (PMC8856522; doi:10.1371/journal.pone.0263366)
Supplement: S1 Table — (PDF) [file pone.0263366.s001.pdf]

S1 Table.

The codebook developed for the thematic analysis.

Theme/ sub-theme	The characteristics of quotes to be categorised in theme.
Consequences of Criticism	
Altered self-perceptions	References to viewing the self negatively. Any self-criticisms. Any reference to attempting to change who they are.
Sensitivity to criticism	References to any negative emotional reactions to criticisms, in the short or long term.
Coping with criticism	
Active change	Any references to altering their environment, including work, friends, or level of social interaction.
Hiding ADHD	References to hiding symptoms or behaviours of ADHD, or not sharing with people that they have a clinical diagnosis of ADHD.

It's not me, its them	Any references to participants viewing criticism as a reflection of other people's opinion, mental health, personality or culture.
Knowing and accepting the self	References to accepting the self, learning about the self, or knowing the self in a way that is beneficial for resilience or coping with criticism experienced now, or in the past.
Openness to criticism	Any references to viewing criticism positively or seeing the worth in criticism.
The role of support and understanding	
Misunderstanding as a precursor to criticism	Any evidence that misjudgement or criticism is the consequence of misunderstanding
The benefits of understanding	Any evidence that understanding has reduced criticism, enhanced support, aided resilience towards criticism, improved relationships with self/others.

Why do others lack understanding?	Any discussion of others not accepting ADHD as a disorder
What is criticised?	
"Everything I do"	Responses that directly state they are criticised for everything. Does not include responses that list numerous criticisms.
Focus and Inattention	Responses that directly, or indirectly state that they are criticized for inattention or a lack of focus. This can be through describing behaviours that represent this (e.g. daydreaming).
Forgetfulness	Responses that directly, or indirectly state that they are criticized for forgetting.
Impulsivity & self-Control	Responses that directly, or indirectly state that they are criticized for engaging in impulsive behaviours or showing little self-control. This can include descriptions of behaviours that result from this e.g. (talking too much).

Organisation	Responses that directly, or indirectly state that they are criticized for poor organisation skills.
Time Management	Responses that directly, or indirectly state that they are criticized for poor time management. This includes being late, missing deadlines, being too slow/fast at tasks.
What is perceived as criticism?	
(Mis)judgement	References to perceiving judgement from others. Any reference to traits that could be explained by underlying ADHD (lazy, scattered, etc.).
Comparisons with others	References to being compared with peers.
Expectations	References to other people's expectations and/or being unable to meet expectations.
Humour	References to using humour to deliver criticism, such as jokes,

	mocking, sarcasm etc.
Others emotional reactions	References to perceiving other people’s reactions towards them and their behaviours negatively.
Rejection	References to being excluded, ignored, being rejected by family/friends.
